# Physical growth during the first year of life. A longitudinal study in rural and urban areas of Hanoi, Vietnam

**DOI:** 10.1186/1471-2431-12-26

**Published:** 2012-03-12

**Authors:** Huong Thu Nguyen, Bo Eriksson, Liem Thanh Nguyen, Chuc Thi Kim Nguyen, Max Petzold, Göran Bondjers, Henry Ascher

**Affiliations:** 1 Research Institute for Child Health, National Hospital of Pediatrics, 18/879 La Thanh Road, Dong Da district, Hanoi, Vietnam; 2 Nordic School of Public Health, PO Box 12133, SE-402 42 Gothenburg, Sweden; 3 Family Medicine Department, Hanoi Medical University, No.1 Ton That Tung Street, Hanoi, Vietnam; 4 Sahlgrenska Academy, University of Gothenburg, PO Box 440, SE-405 30 Gothenburg, Sweden

## Abstract

**Background:**

Good infant growth is important for future health. Assessing growth is common in pediatric care all over the world, both at the population and individual level. There are few studies of birth weight and growth studies comparing urban and rural communities in Vietnam. The first aim is to describe and compare the birth weight distributions and physical growth (weight and length) of children during their first year in one rural and one urban area of Hanoi Vietnam. The second aim is to study associations between the anthropometric outcomes and indicators of the economic and educational situations.

**Methods:**

Totally 1,466 children, born from 1^st ^March, 2009 to June 2010, were followed monthly from birth to 12 months of age in two Health and Demographic Surveillance Sites; one rural and one urban. In all, 14,199 measurements each of weight and length were made. Birth weight was recorded separately. Information about demographic conditions, education, occupation and economic conditions of persons and households was obtained from household surveys. Fractional Polynomial models and standard statistical methods were used for description and analysis.

**Results:**

Urban infants have higher birth weight and gain weight faster than rural infants. The mean birth weight for urban boys and girls were 3,298 grams and 3,203 grams as compared to 3,105 grams and 3,057 grams for rural children. At 90 days, the urban boys were estimated to be 4.1% heavier than rural boys. This difference increased to 7.2% at 360 days. The corresponding difference for girls was 3.4% and 10.5%. The differences for length were comparatively smaller. Both birth weight and growth were statistically significantly and positively associated with economic conditions and mother education.

**Conclusion:**

Birth weight was lower and the growth, weight and length, considerably slower in the rural area, for boys as well as for girls. The results support the hypothesis that the rather drastic differences in maternal education and economic conditions lead to poor nutrition for mothers and children in turn causing inferior birth weight and growth.

## Background

Growth of children is influenced by maternal, environmental, genetic and hormonal factors. Nutrition is assumed to be the one of the most important factors for the growth of infants [1]. Some reasons for growth failure in children could be problems in child well-being as well as underlying chronic illnesses or inadequate nutrition [2]. Slow intrauterine and infant growth can influence the weight gain in childhood and later in life increase the risk for diseases like coronary heart disease, type 2 diabetes and hypertension [3]. Assessing growth, both at population and individual level, is common in pediatric care all over the world. At a population level, growth assessment of children means estimating prevalence of undernourishment, overweight and identification of different groups in need of intervention [4]. Differences in birth weight and growth of children between urban and rural areas have been reported in some studies [5-8]. Inequality of family income, general living conditions, average number of children in families and nutrition have been pointed out as the main explanations for such differences [6,7].

At the individual level, children are followed over time. Growth of the single child is compared to a growth chart, which is a diagram showing standard weight for age, length or height for age, weight for height and other anthropometric measures as functions of child age. The graphic description most often includes mean with standard deviations as functions of child age, or in the case of weight, of length or height. This follow-up is used to evaluate deviations of the growth in individual children which could be early signs of ill-health.

In Vietnam there has been a dramatic improvement in economic conditions since the Doi Moi reforms starting in 1986; income per capita has increased from $130 to $900 from the early 1990s until 2008. Absolute poverty has been reduced from 58 percent of the population in 1993 to 13 percent in 2008 [9]. The prevalence of underweight of children has decreased from 45% in 1990 to 26.6% in 2004. The rate of reduction of malnutrition has been higher in urban areas than in rural areas [10]. The percentage of low birth weight in Vietnam was estimated to be higher in rural areas (5.9%) than in urban areas (3.9%) in 2002 [11]. Over the last decades, a few longitudinal studies of rather small groups were conducted to follow the growth of children born in delivery clinics or maternal hospitals [12-14]. Generally, however, there is a lack of knowledge about birth weight and growth of larger groups of children as well as comparisons between urban and rural communities of Vietnam.

A hypothesis is that birth weight is lower and that growth is slower in the rural area due to different nutritional conditions that could in turn be related to economic resources and education. The first aim of this study is to describe and compare the birth weight distributions and physical growth (weight and length) of children from birth to 12 months in one rural and one urban area of Hanoi, Vietnam. A secondary aim is to study associations between the anthropometric outcomes and variables indicating the economic and educational situations.

## Methods

### Study sites

The study was conducted in two Health and Demographic Surveillance Sites (HDSS), one urban and one rural, in Hanoi, the capital of Vietnam. Dongda is an urban district in central Hanoi with about 352,000 inhabitants. Three communes, among 21, in the district, were strategically selected to have different economic levels. In each commune a representative ward was selected. The populations of these, totally close to 40,000 persons in 11,500 households, were defined as the DodaLab HDSS in 2007 [15]. Bavi is a rural district, also within Hanoi with 250,000 persons. About 52,000 persons in 13,000 households situated in 69 randomly selected clusters out of 352 called FilaBavi HDSS, have been followed since 1999 [16].

Household surveys were undertaken in both sites during late 2007 and 2008 as well as during 2009 to obtain information about demographic conditions, education, occupation and economic conditions of persons and households. In both sites, all households are routinely visited every three months to record vital events, birth, death, migration and pregnancies.

### Study design and subjects in the follow-up of child growth

The parents of all children reported to have been born alive from 1^st ^March, 2009 to 30^th ^June, 2010, in DodaLab and FilaBavi, were invited to enroll their child in the study. Children with congenital and malformation diseases (two in DodaLab and six in FilaBavi) were not invited. About 1%, totally 15 with 12 in DodaLab, of the mothers did not give consent and the child was not enrolled. Altogether 12 children were born as twin and were not used in the present analysis. Low birth weight infants (below 2,500 g) were included in the analysis, since their growth potential was considered as normal [17]. The measurements made on later out-migrated (61 from DodaLab and 27 from FilaBavi) or children who died (altogether five, four of them in DodaLab) have been used in the analysis.

Totally 1,466 children were used to analyze growth during the first year of life. The 540 DodaLab children provided 4,964 measurements each of weight and length. In FilaBavi 926 children contributed with 9,235 measurements. Totally 14,199 measurements were analyzed, that is 9.7 measurements per child.

### Measurements and data collection

Birth weight information was provided by the mothers, who reported the measurement made at the hospital or commune health centers immediately after birth. For less than 1% of the children birth weight information was not obtained. The information about birth weight has been analyzed separately from the subsequent measurements of growth.

Given the mother's consent, children were registered for the study and scheduled for measurement of weight and length every month from one month after birth to the age of 12 months. The percentages of scheduled measurements actually done were 65% for DodaLab and 77% for FilaBavi. The frequency of missed measurements increased with the age of the infant. The percentage of children actually followed to at least 11 months was 80% in DodaLab and 90% in FilaBavi.

Standardized equipment for measuring the child recommended from Hanoi Medical University was used. A number of commune health centre staff members in DodaLab were trained specifically to measure children. In FilaBavi, a number of the permanent interviewers were trained to measure children. The principle of measurement was that the same field worker should assess a child at each visit using the same equipment. Weight was measured to the nearest 10 gram with the child in light clothes using a portable infant scale. Length was measured to the nearest centimeter in horizontal position using a length board. Two person worked together in order to have valid and reliable measurements [12].

The difference between the date of birth and the date for the last menstruation reported by the mother can be assumed to be correlated to the gestational age at birth. In spite of the likely underestimation of the true pregnancy time the difference is used as a proxy for the gestational age and will subsequently be referred to as the Gestational age proxy (Gap).

Data describing economy and education were taken from the household surveys conducted 2009 in the two sites. At household level we considered the reported yearly household income and the household assets available (according to a specified list) as indicators of economic resources. The number of household members was also studied.

For the mothers we studied age and education (primary, secondary and higher). In the urban area the dominant occupational category was office and service employment. Farming was the most frequent occupation in the rural area. However, occupation is strongly correlated to education and has not been used in the analysis.

### Statistical analysis

Assessments of associations between the dependent variable birth weight and the independent area, sex, mother's age, education occupation, reported household income and sum of household assets were made using linear regressions. No distinction of term or preterm children was made but the Gap indicator was used as an indicator of gestational age.

The statistical description of weight and length growth has two objectives, the estimation of mean and variation of attained weight and length as functions of child age and the corresponding growth velocity also as a function of child age. Theoretically the velocity functions are the first derivatives of the attained weight and length functions.

Several methods have been suggested for statistical description and analysis of growth data [18]. The ambition for the present work was to use a simple approach, still theoretically and scientifically defendable. Some different models for smoothing curves were tried. The finally selected were Fractional Polynomial Models [19] which provided good fit with reasonably simple forms. The study of residuals in the weight model (not for length) suggested that a logarithmic transformation should improve normality. The models presented therefore are Fractional Polynomials of degree 2 with relative residuals assumed to be normally distributed with constant variance, in the case of weight after logarithmic transformation. Subgroup specific fitted Fractional Polynomials were used to describe the growth by area and sex.

Differences in growth between the sites and child sex and other independent variables were assessed using two- level, mixed effect linear models. The dependent variables were the relative residuals (logarithmic for weight) from the overall fitted Fractional Polynomials.

The deviations from the WHO standard curves were evaluated for statistical significance using the child specific means of relative deviations from the standards.

Growth velocity was calculated as the first derivative of the fitted fractional polynomials.

In addition the average growth velocity for each child over the first year, obtained through collapsing the dataset to child level was used.

Three linear regression models were used for the analysis of birth weight and residuals from the growth curve:

Model A. independent variables: area (urban vs. rural), Gap and child sex.

Model B. independent variables: area (urban vs. rural), Gap, child sex, education and household assets.

Model C. independent variables: area (urban vs. rural), child sex, Gap, education, household assets, mother age, household income and number of household members.

The software used for all analysis was STATA version 11. In the analysis we used only singleton children.

### Ethical consideration

Approval of the project was obtained from the Scientific and Ethical Committee of Hanoi Medical University, Hanoi Health Bureau and Dongda district authorities. The proposal was approved by the Ministry of Health and permission for the study was given after the baseline survey. All mothers of infants were informed about the purpose of the studies and their right to decline participation or withdraw. Consent for participation was given by all mothers of the included infants.

## Results

### Birth weight

Wide and highly statistically significant differences in mean birth weight were found between the urban and rural areas. Table 1 shows means, standard deviations and confidence intervals by area and child sex. The distribution of the birth weights reported by the mothers was reasonably symmetric. The estimated birth weight difference between the areas for boys was 193 g (95% CI: 134; 252) and for girls 146 g (95% CI: 79; 213). The mean birth weight of the urban girls was actually significantly higher than of the rural boys (p < 0.01).

**Table 1:** Birth weight and background variables

	Urban boys	Urban girls	Rural boys	Rural girls
Birth weight,	3298	3203	3105	3057
mean standard deviation	450	435	390	408
and 95% confidence interval, grams	(3263, 3422)	(3148, 3259)	(3071,3139)	(3017, 3097)
Low birth weight, %	2.3	4.2	4.1	4.9
Number of children in study	300	237	513	409
Days from reported last menstruation to birth, mean	272	271	271	272
Mother age, mean, years	28.7	28.3	25.4	25.3
Mother' highest education primary school, %	8.6	4.9	54.8	54.6
Mother education higher than secondary school, %	58.2	67.1	17.4	16.8
Number of household members, mean	4.6	4.4	5.2	5.7
Number of household assets, mean	9.4	9.1	4.7	4.8
Yearly household income, median million VND	75 300 000	78 600 000	35 000	35 000

Table 1 also gives an overview of the variables that have been considered as independent variables in the regression models i.e. area (urban vs. rural), child sex, Gap, mother age, mother education (three levels), household income, number of household members and number of household assets. A key feature of this information is that rural mothers are younger and less educated than the urban. The reported number of assets and income are higher in urban households, drastically so for income. The household size is larger in the rural area.

Table 2 shows the regression results. In model A and B, area, child sex and Gap variables exhibit low p-values but the regression coefficient for area decreases markedly. This tendency continues into Model C where the coefficient is very low and accompanied with a high p-value. The negative sign of the regression coefficient for education in the birth weight analysis is due to the difference in distribution between the urban negatively skewed and the rural positively skewed distributions.

**Table 2:** Regression analysis of birth weight

	Model A		Model B		Model C	
	Regression coefficient	p-value	Regression coefficient	p-value	Regression coefficient	p-value
Area urban-rural	179	0.000	158	0.000	20	0.877
Child sex						
Boys-girls	63	0.003	61	0.004	60	0.006
Gestational age proxy	4.4	0.000	4.3	0.000	4.3	0.121
Mother age					2.1	0.366
Education			-24	0.133	-25	0.121
Assets gram incr. per item			10	0.022	8.5	0.083
Income (logarithm)					17	0.309
Household members					-5.2	0.301
Explanatory value R^2^	R^2 ^= 0.0897		R^2 ^= 0.0942		R^2 ^= 0.0976	

### Infant growth

**Figure 1:**
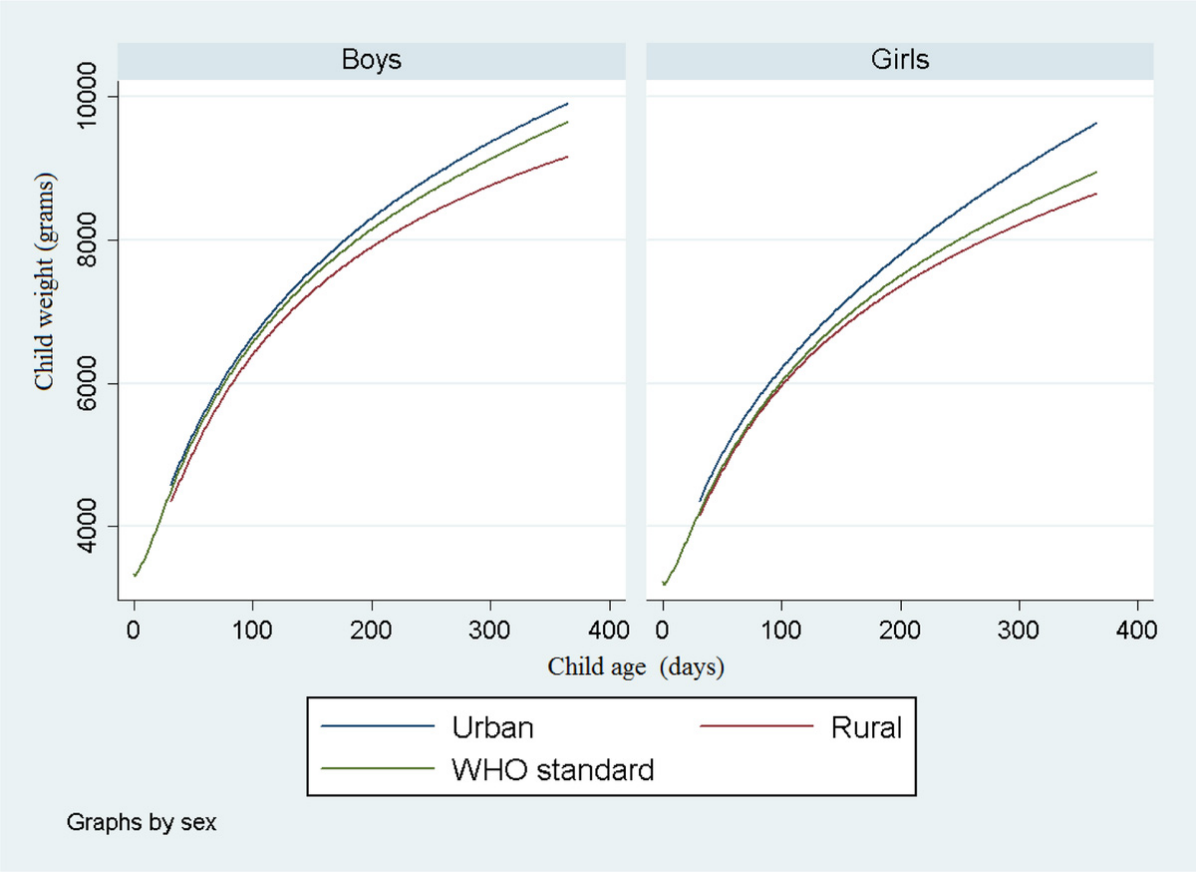
**Estimated mean curves for attained weight for age by sex together with WHO standard**.

**Figure 2:**
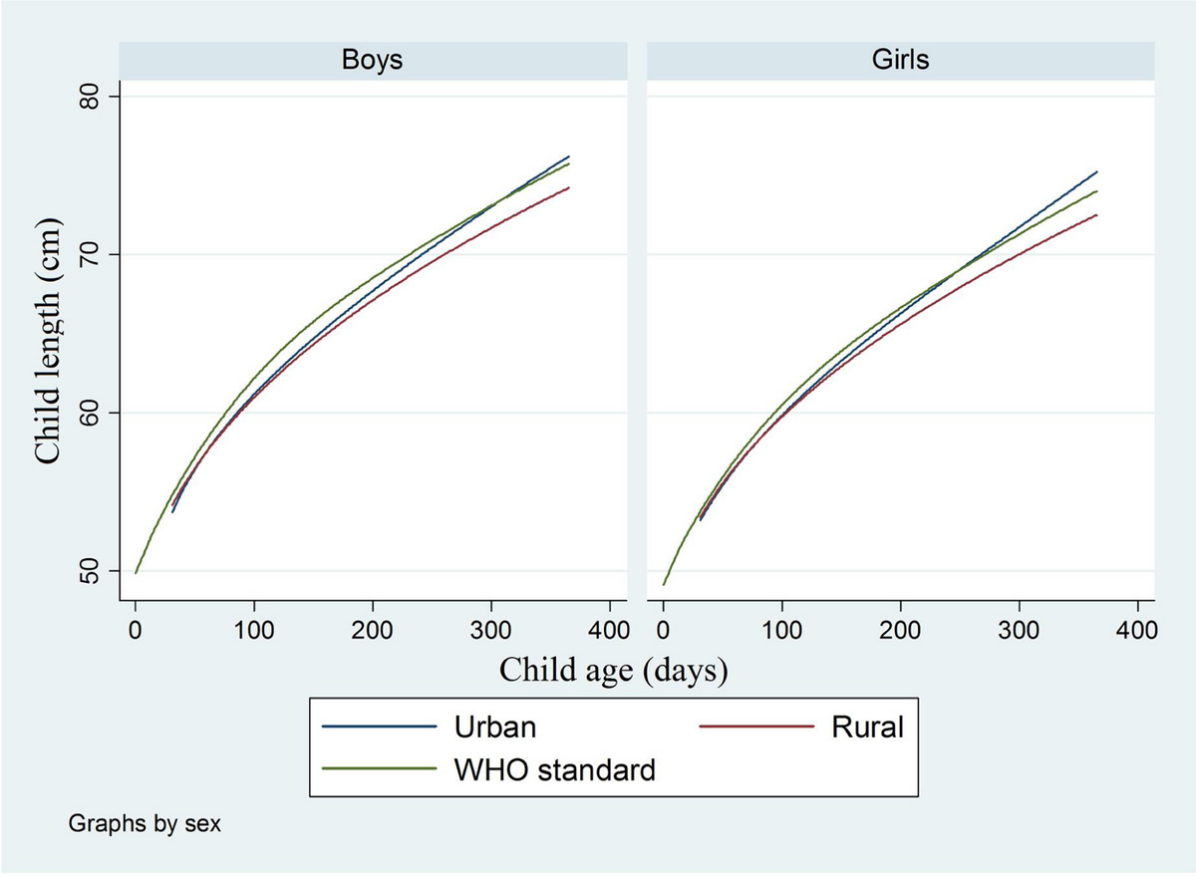
**Estimated mean curves for attained length for age by sex together with WHO standard**.

The estimated growth curves differed statistically significantly between the sites for both sexes (Figure 1). The mean attained weight was generally higher in the urban area than in the rural and, as seen in the graph, increased in absolute term with increasing age. The p-values from the two-level analysis of residuals were smaller than 0.001 both for the area and the child sex comparison. The same tendencies and p-values were seen for the mean attained length (Figure 2).

Lines showing the WHO growth standards published in 2006 [20] are included in Figures 1 and 2. The WHO curve for weight falls between the fitted curves for the urban and the rural area for both child sexes. The deviations from the WHO standard are statistically significant in all cases (p < 0.01). The WHO standard for length is significantly higher for the rural area (p < 0.01). For the urban no significant deviation can be stated.

Estimated attained weight (grams) and limits for plus and minus two standard deviations at 90, 180, 270 and 360 days of age differed between the two sites (Table 3). The differences of infant growth in weight between urban and rural areas increased with increasing age. At 90 days, the urban boys were estimated to be 4.1% heavier than the rural boys. This ratio increased to 7.2% at 360 days. The corresponding numbers for girls were 3.4% and 10.5%. Urban girls were almost 0.5 kg heavier than rural boys at one year of age. The asymmetry of the limits is due to the residual skewness.

**Table 3:** Attained weight (grams) with limits for plus and minus two standard deviations at selected ages

	Urban area				Rural area			
	**Boys**		**Girls**		**Boys**		**Girls**	

**Age**	**Mean**	**(± 2 SD)**	**Mean**	**(± 2 SD)**	**Mean**	**(± 2 SD)**	**Mean**	**(± 2 SD)**

90 days	6432	(5176,7992)	5999	(4703,7652)	6166	(4970,8562)	5794	(4646,7112)
180 days	8037	(6468,9986)	7541	(5912,8517)	7688	(6198,9490)	7156	(5783,8851)
270 days	9066	(7296,11264)	8618	(6757,9734)	8521	(6870,10568)	7982	(6451,9874)
360 days	9894	(7963,12294)	9644	(7561,12301)	9173	(7395,11377)	8624	(6970,10668)

The estimated attained length (cm) and limits for plus and minus two standard deviations at 90, 180, 270 and 360 days of age also differed between the two sites (Table 4). The residual distributions for length were symmetrical and thus also the standard deviation limits.

**Table 4:** Attained length (cm) with limits for plus and minus two standard deviations at selected ages

	Urban area				Rural area			
	**Boys**		**Girls**		**Boys**		**Girls**	

**Age**	**Mean**	**(± 2 SD)**	**Mean**	**(± 2 SD)**	**Mean**	**(± 2 SD)**	**Mean**	**(± 2 SD)**

90 days	60.3	(54.6,66.0)	59.1	(53.4,64.8)	60.1	(55.0,65.2)	59.0	(54.3,63.8)
180 days	66.5	(60.2,72.9)	65.1	(58.9,71.3)	66.1	(60.5,71.8)	64.6	(59.4,69.8)
270 days	71.5	(64.7,78.3)	70.1	(63.4,76.8)	70.5	(64.4,76.5)	68.8	(63.3,74.3)
360 days	76.0	(68.8,83.2)	75.0	(67.8,82.2)	75.9	(67.6,80.2)	72.3	(66.5,78.1)

Estimated weight growth velocity and length growth velocity at 90, 180, 270 and 360 days decreased throughout the first year of life in both sites (Table 5). The differences of growth velocity between the rural and urban infants increased over age. This was particularly evident for the weight differences at all ages. Table 5 also shows growth velocity in the first year of life with confidence limits. The rural area estimates are significantly lower than the urban for growth velocity in weight (p < 0.05). For length, rural girls grow significantly slower than the other groups (p < 0.05).

**Table 5:** Growth velocity at selected ages and average velocity from 90 to 360 days with 95% confidence intervals

	Weight (gram/day)				Length (cm/10 days)			
	**Urban**		**Rural**		**Urban**		**Rural**	

**Age**	**Boys**	**Girls**	**Boys**	**Girls**	**Boys**	**Girls**	**Boys**	**Girls**

90 days	24.0	20.9	23.3	21.4	.84	.82	.79	.76
180 days	14.1	13.8	12.2	11.4	.60	.56	.59	.59
270 days	10.0	11.5	7.8	7.7	.51	.43	.53	.42
360 days	7.7	10.3	5.7	5.8	.47	.35	.53	.36
Average	12.8	13.5	11.1	10.5	.58	.59	.59	.49
Lower limit	12.2	12.7	10.7	9.9	.56	.57	.57	.48
Upper limit	13.4	14.3	11.6	11.1	.60	.61	.60	.50

The associations between growth and the independent variables described in Table 6 show the regression coefficients and p-values for Model A and C analysis of the mean relative residuals for attained weight and length. The results are significant and similar to those of birth weight child sex, Gap, household assets and education. The area variable association changes with the complexity of the model as for birth weight. For length, only the child sex and Gap variables are statistically significant.

**Table 6:** Regression analysis of residuals from growth curves

	Model A				Model C			
	Weight		Length		Weight		Length	
	**Coeff**	**p**	**Coeff**	**p**	**Coeff**	**p**	**Coeff**	**p**

Area	.057	.000	.010	.000	.044	.157	-.012	.292
urban- rural								
Child sex	.061	.000	.021	.000	.060	.000	.020	.000
Boys-girls								
Gestational age proxy	.00041	.002	.00014	.003	.00041	.003	.00001	.011
Mother age					-.00006	.917	.0002	.238
Education					.010	.010	.0024	.125
Assets gram incr. per item					.0029	.010	.00072	.106
Income (logarithm					-.0010	.802	.0022	.191
Household members					.00002	.988	-.0007	.162
Explanatory value R^2^	.1523		.0857		.1674		.0995	

## Discussion

The main findings of the study are the differences between urban and rural areas in birth weight as well as in the subsequent growth, attained weight and length and growth velocity. For birth weight the differences between boys and girls were expected as was also the associations with the gestational age proxy. The latter is the variable with the strongest correlation to birth weight and is in turn related to subsequent attained weight and length.

The area variable in itself, urban vs. rural, is of no importance when other variables, with large differences between the areas, are introduced in Model C. Some of the added variables are not statistically significantly associated to birth weight or growth but obviously form an intricate pattern that "replaces" the area variable. This finding is the same in the analysis of birth weight and in the analysis of growth. Another common finding is that there are associations between growth and household assets and education, particularly for weight growth.

Growth velocity for weight differs between the areas for both child sexes. The length growth velocity is lower for rural girls. It shall be noted that all regression models have quite low values for the determination coefficient (R^2^) and that the largest part is contributed by the area and sex variables meaning that rather small fractions of the variation in birth weight and growth are explained by the associations with Gap, area and child sex differences and the social and economic variables.

The result from the present study is in accordance with results from previous studies in other countries [6,7,21]. Differences in growth of infants between urban and rural areas have been described in Peru in 1980. Height for age and weight for age of rural infants did not catch up to urban infants [21]. Newer studies in China show that urban infants grow faster than rural infants [6,7].

Socioeconomic conditions, nutrition of mothers during pregnancy, antenatal care, and increased maternal weight gain during pregnancy have been seen to be associated to the birth weight of the child [22-25]. Economic advantages, better education can lead to better nutrition for mothers and faster fetal weight gain. A Vietnamese study in 1996 found that 94% of rural farming women had insufficient food intake, compared to 40% for non- farming women [26]. This situation has improved, but there can still be considerable differences in food intake between farming and non-farming women in Vietnam. The prevalence of anemia in women was higher in a rural area than in an urban in India [27]. In Vietnam, no results on the prevalence of anemia in urban areas are available but a study in 2005 reported that in a rural area the prevalence among pregnant women was as high as 43.2% [28].

The rural mothers of the children in the present study attended antenatal care (ANC) later, had fewer visits and much less of specific medical services than in the urban mothers [15]. Differences in antenatal care could be one factor behind the differences found in birth weight and infant growth. Specifically poor adherence to the guidelines for medical services can mean that conditions disadvantageous for growth are not detected.

Several conditions and factors have been shown as associated to poor growth of infants with nutrition as the most important [1,29]. The nutritional status of under five children is proposed as a sensitive indicator of economic condition [30]. Some studies therefore explain differences in child growth between rural and urban areas with differences in family income and general living conditions. Fewer children in the urban families might lead to better nutrition of each child [6,7]. Parent's education has been demonstrated to be one of the main contributing factors for under five malnutrition in Bangladesh [30].

In Vietnam, the total fertility rate in the rural areas was higher than in the urban area [31] but the income per capita in urban areas was higher than in the rural [15,16] Maternal education was also higher in the urban area than in the rural. Both economy and education might contribute to a better nutritional situation for infants in urban areas. The present study shows drastic differences in the educational and economic situation between the urban and rural mothers and households. There is also a tendency to smaller households in the urban area.

The differences in weight gain between rural and urban infants found in this study are established at an early age. One important factor may be differences in breastfeeding patterns, especially the duration of exclusive breastfeeding. The absolute differences in growth of infants between urban and rural areas increased with increasing ages. Use of different types of supplement food for infants in the two sites could explain this.

Infants in the urban area are likely to have easier access to child health care than rural infants. Some barriers to access child health care in rural areas in Vietnam, like distance and long travel times, do exist. Financial, sociocultural, language, ethnicity are other possible barriers together with lack of knowledge, awareness and inequalities in quality of health care [32].

The differences of length growth between the two sites were comparatively smaller at low ages, but increased in absolute terms during infancy. This result is in agreement with results of studies from China where urban children were taller than rural children at all ages from one to 12 months of age [6,7]. One study found that the difference of growth in length of children between rural and urban areas is statistically significant only after six months and especially after 2 years of age [6].

Different standards for child growth have been published by various institutions and international organizations. Recently, the World Health Organization (WHO) launched growth standards in 2006. These were constructed to show child growth under ideal conditions [20]. A study in Vietnam that assessed the growth of children by using the new WHO child growth standards as reference showed that deficient growth of infant is widespread in Vietnam [14]. Another study in an urban area of Hanoi found that the growth of Vietnamese infants was also lagging behind the earlier used National Centre for Health Statistic reference population [13]. The present results put urban boys and girls above the WHO standards and the rural children below for weight. For length again the rural curves are below the standard. This can be seen as an indication that genetic factors could not explain deviations in weight growth at a population level in Vietnamese infants. A detailed analysis of the relation between the present results and the WHO standards is beyond the scope of this paper but further analysis seems urgent, not at least to explore when early signs and warning of subsequent overweight can be detected.

Compared to results of a study in urban Hanoi in the 1990's, the birth weight and growth of infants in the present study are higher for both sites [13], indicating that the birth weight and growth of infants in both rural and urban areas of Hanoi have improved. There is, however, still a gap between the rural area and the urban area suggesting differences in child health care and nutrition.

One limitation of the study is the short follow-up time. One year is not enough to study if differences tend to decrease or increase as the children get older. The ambition for further research shall be to continue follow-up to at least 5 years to see if the rural children catch up with urban children or if the gaps are further widened. Also the exploration of overweight tendencies will require longer follow-up. Certain unavoidable differences between the study designs, data collection and administrative procedures might be seen as limitations. For example the two cadres of interviewers have different employment conditions. But the good training and the quality control have probably minimized this problem. The situation that there are unequal sample sizes in the two areas is not optimal for comparison.

The research was conducted in two sites within the capital of Vietnam. These areas are generally considered to have rather good socioeconomic conditions compared to the rest of country. Even so, the birth weights and growth of infants are higher in the urban area than in the rural area. This suggests that differences are likely to occur also in other, comparatively poorer, settings in Vietnam.

## Conclusion

Mean birth weight as well as weight growth of infants, described both as attained weight at different ages and growth velocities were different between the investigated areas in Vietnam. The birth weight was lower and the growth considerably slower in the rural area, for boys as well as for girls. The corresponding differences in length growth of the infants were more modest but increased with age during the first year of life. The results support the hypothesis that the rather drastic differences in mother education and economic conditions leads to poor nutrition for mothers and children in turn causing inferior birth weight and growth. The importance of health care utilization and breastfeeding are two areas that will need further exploration.

## Competing interests

The authors declare that our findings have not been influenced by our personal or financial relationship with other person or other organization.

## Authors' contributions

HNT led and supervised the fieldwork and data management. She also drafted and completed this paper. BE assisted in the research design as well as in the statistical analyses, interpretation of results and revising the manuscript. HA, LNT, CNTK, MP and GB were involved in the design of the study, supervised the study and revised the manuscript. All authors have read and approved the final manuscript.

## Authors' information

Huong Nguyen Thu MD, researcher and pediatrician of the Research Institute of Child Health and the National Hospital of Pediatrics in Hanoi, Vietnam. She is also a PhD student of the Nordic School of Public Health in Gothenburg, Sweden

Bo Eriksson PhD, Professor emeritus of the Nordic School of Public Health in Gothenburg, Sweden

Liem Nguyen Thanh MD, PhD, Professor and Director of the Research Institute of Child Health and the National Hospital of Pediatrics in Hanoi, Vietnam

Chuc Nguyen Thi Kim PhD, Associated professor of Hanoi Medical University

Max Petzold is PhD, Professor of the Nordic School of Public Health and the Gothenburg University in Sweden

Göran Bondjers MD, PhD is Professor of Gothenburg University in Sweden

Henry Ascher MD, PhD, Associate professor of Nordic School of Public Health, Gothenburg, Sweden

## Pre-publication history

The pre-publication history for this paper can be accessed here:

http://www.biomedcentral.com/1471-2431/12/26/prepub
